# Peroxidation rate constants and mechanisms of isoprenoid-derived lipids and their roles in ferroptosis

**DOI:** 10.1016/j.rbc.2025.100058

**Published:** 2025-08-22

**Authors:** Noelle Reimers, Libin Xu

**Affiliations:** Department of Medicinal Chemistry, University of Washington, Seattle, WA, 98195, USA

## Abstract

Ferroptosis is a type of regulated cell death that is dependent on iron and driven by lipid peroxidation. Polyunsaturated fatty acids (PUFAs) sensitize cells to ferroptosis as they are prone to lipid peroxidation while monounsaturated fatty acids confer resistance to ferroptosis when incorporated into the lipid membrane as they are much less reactive toward lipid peroxidation. Recently, in addition to fatty acid-derived lipids, isoprenoid-derived lipids have been found to regulate ferroptosis. Specifically, ferroptosis suppressor protein 1 (FSP1) was found to be anti-ferroptotic as it reduces the oxidized forms of coenzyme Q_10_ and vitamin K to their reduced quinol forms, which are phenolic radical-trapping antioxidants. Vitamins D_3_ and A have also been found to inhibit ferroptosis in cancer cells. Furthermore, it has been shown that metabolites along the cholesterol synthesis pathway, including squalene, cholesterol, desmosterol, and 7-dehydrocholesterol (7-DHC), can protect cells against ferroptosis in vitro. Despite large variations in the reactivities of these lipids toward lipid peroxidation, they generally exhibit anti-ferroptotic properties. In this review, we will discuss the peroxidation rate constants and mechanisms of these isoprenoid-derived lipids and how they might contribute to their roles in ferroptosis.

## 1. Introduction

Ferroptosis is a type of regulated cell death first characterized by the Stockwell lab in 2012 [1]. It is dependent on the iron-mediated peroxidation of polyunsaturated fatty acid- (PUFA)-containing phospholipids in cell membranes, which results in membrane permeabilization and eventual cell death [1–5]. It has been implicated in many pathological conditions, including tissue injury, degenerative diseases, and cancer [6–8]. Therefore, inhibiting or promoting ferroptosis holds significant therapeutic potential.

Inhibition of glutathione peroxidase 4, or GPX4, is the canonical method used to induce ferroptosis in cells [2,9]. GPX4 is a membrane-bound peroxidase enzyme that uses glutathione to reduce lipid peroxides into nontoxic lipid alcohols. It can be inhibited directly with the compound RSL3, which was discovered in a cancer drug screening in the Stockwell lab in 2008 [10]. It can also be inhibited indirectly by glutathione depletion. This is commonly achieved using the compound erastin, which inhibits system ${\text{X}}_{\text{c}}^{-}$ mediated cystine import [1,11].

In recent years, different mechanisms of ferroptosis regulation that depend on lipid metabolism have been elucidated. It has been shown that ACSL4 is essential to ferroptotic death in many cell lines due to its role in incorporating polyunsaturated fatty acids (PUFAs) into phospholipids [4]. The mitochondrial flavoprotein enzyme that was previously known as AIFM2 was renamed to ferroptosis suppressor protein 1 (FSP1) for its role in regenerating ubiquinol from ubiquinone, which prevents lipid peroxide buildup in membranes [12,13]. Recently, it has been shown that metabolites along the cholesterol synthesis pathway, including cholesterol, desmosterol, 7-dehydrocholesterol (7-DHC), and squalene, can have a protective effect against ferroptosis in vitro [14–21]. Furthermore, other isoprenoids, such as vitamin D_3_ and A, have also been found to inhibit ferroptosis [20]. Therefore, isoprenoid-derived lipids are emerging as key players in mediating ferroptotic death. The peroxidation rate constants of these lipids vary in a wide range, but they generally display anti-ferroptosis properties. We will discuss the structure-reactivity relationship of these lipids and how the different peroxidation rate constants and mechanisms might play a role in ferroptosis.

## 2. Peroxidation rate constants of isoprenoid-derived lipids

**Fig. 1.**
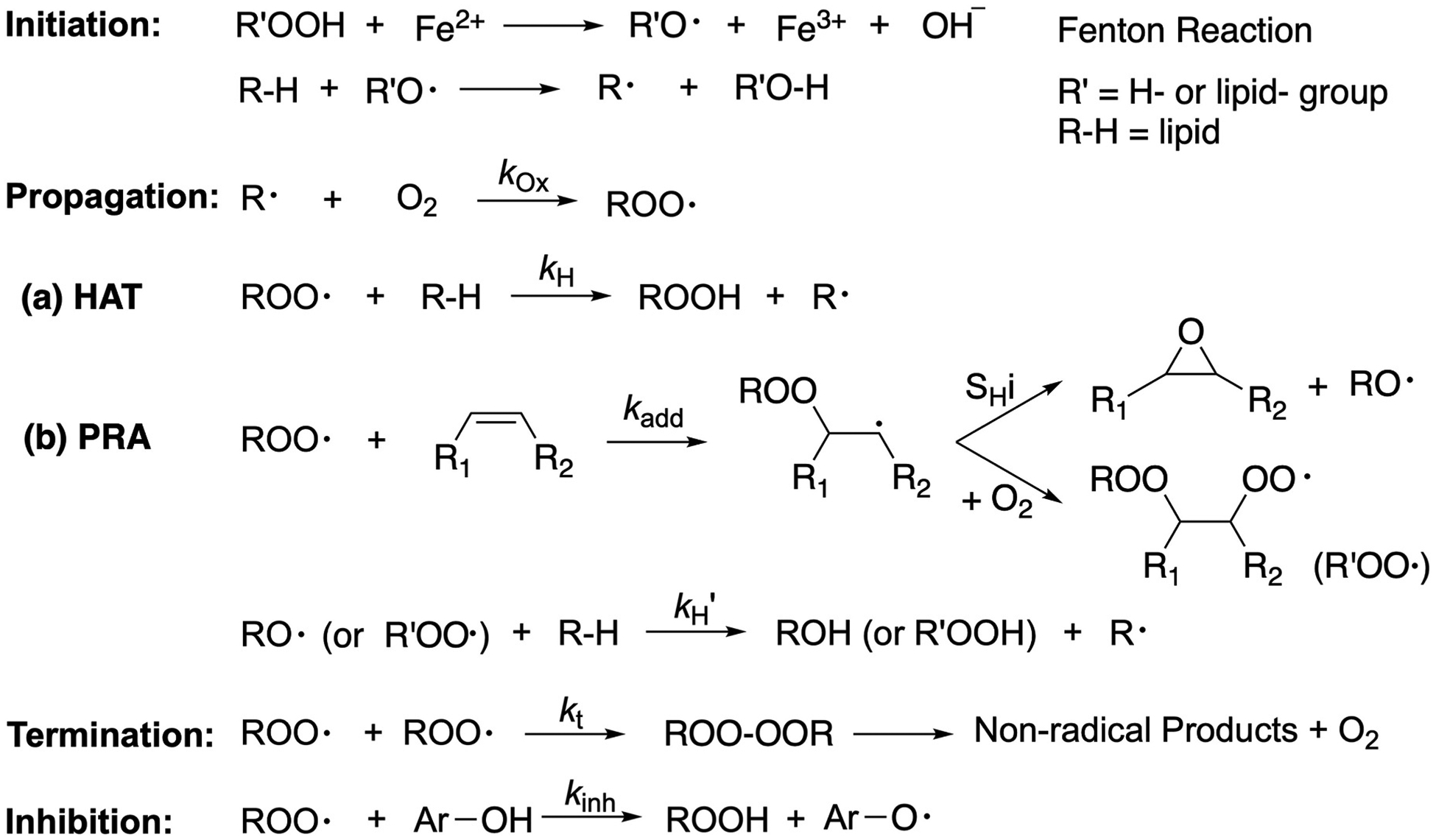
Typical sequence of free radical-mediated lipid peroxidation. Initiation occurs through seeding hydroperoxide and the Fenton reaction. Propagation occurs through hydrogen atom transfer (HAT) and peroxyl radical addition (PRA) reactions of peroxyl radical. Termination occurs when two radical species react with each other, forming non-radical products. Inhibition occurs when a highly reactive hydrogen-atom donor reacts with the peroxyl radical, forming a stabilized radical that does not readily propagate the chain reaction. *k*_Ox_: rate constant of oxygen addition, *k*_H_: rate constant of HAT, *k*_add_: rate constant of PRA, *k*_t_: rate constant of termination, *k*_inh_: rate constant of inhibition.

The chemical reactivity of sterols and other isoprenoid-derived lipids likely dictates their roles in mediating ferroptosis. In biological environments, lipid peroxidation can be initiated by the abstraction of a reactive hydrogen atom from a lipid molecule by seeding radicals, such as alkoxyl or hydroxyl radicals, which can be formed from Fenton reactions between iron and hydrogen peroxide or lipid peroxides (Fig. 1). The seeding lipid hydroperoxides can be formed from reactions catalyzed by lipoxygenases [3,5]. The lipid-derived carbon radicals then react with molecular oxygen to give a peroxyl radical, which propagates the chain reaction. The propagation step can proceed via two mechanisms: hydrogen atom transfer (HAT) and peroxyl radical addition (PRA) (Fig. 1) [22,23]. HAT proceeds with the abstraction of a hydrogen atom from an oxidizable lipid molecule. The resulting carbon radical reacts with molecular oxygen at a diffusion-controlled rate to give a peroxyl radical, which then continues propagating the chain reaction. PRA proceeds with the addition of a peroxyl radical directly to a C=C double bond, often in a conjugated system containing two or more C=C double bonds. The resulting carbon radical can undergo either intra-molecular homolytic substitution (S_H_i) to give an epoxide and an alkoxyl radical or add another molecular oxygen to give a new peroxyl radical. Both the alkoxyl and the peroxyl radical can continue propagating the chain reaction. The rate constants of HAT (*k*_H_) and PRA (*k*_add_) can be measured using two generations of linoleate radical clocks [22,24], which take advantage of the isomerization between *trans*,*cis*- and *trans*, *trans*-dienyl peroxyl radicals resulting from linoleate peroxidation. The overall rate constant of peroxidation is denoted as *k*_p_, which is the sum of *k*_H_ and *k*_add_. On the other hand, endogenous phenolic compounds, such as vitamin E and reduced form of coenzyme Q_10_, can act as radical-trapping antioxidants because they are excellent H-atom donors and the resulting aroxyl radicals do not readily propagate the chain reactions due to their stability. Mechanisms and governing factors of each step of the free radical lipid peroxidation sequence have been discussed and reviewed previously [22,25–28].

**Fig. 2.**
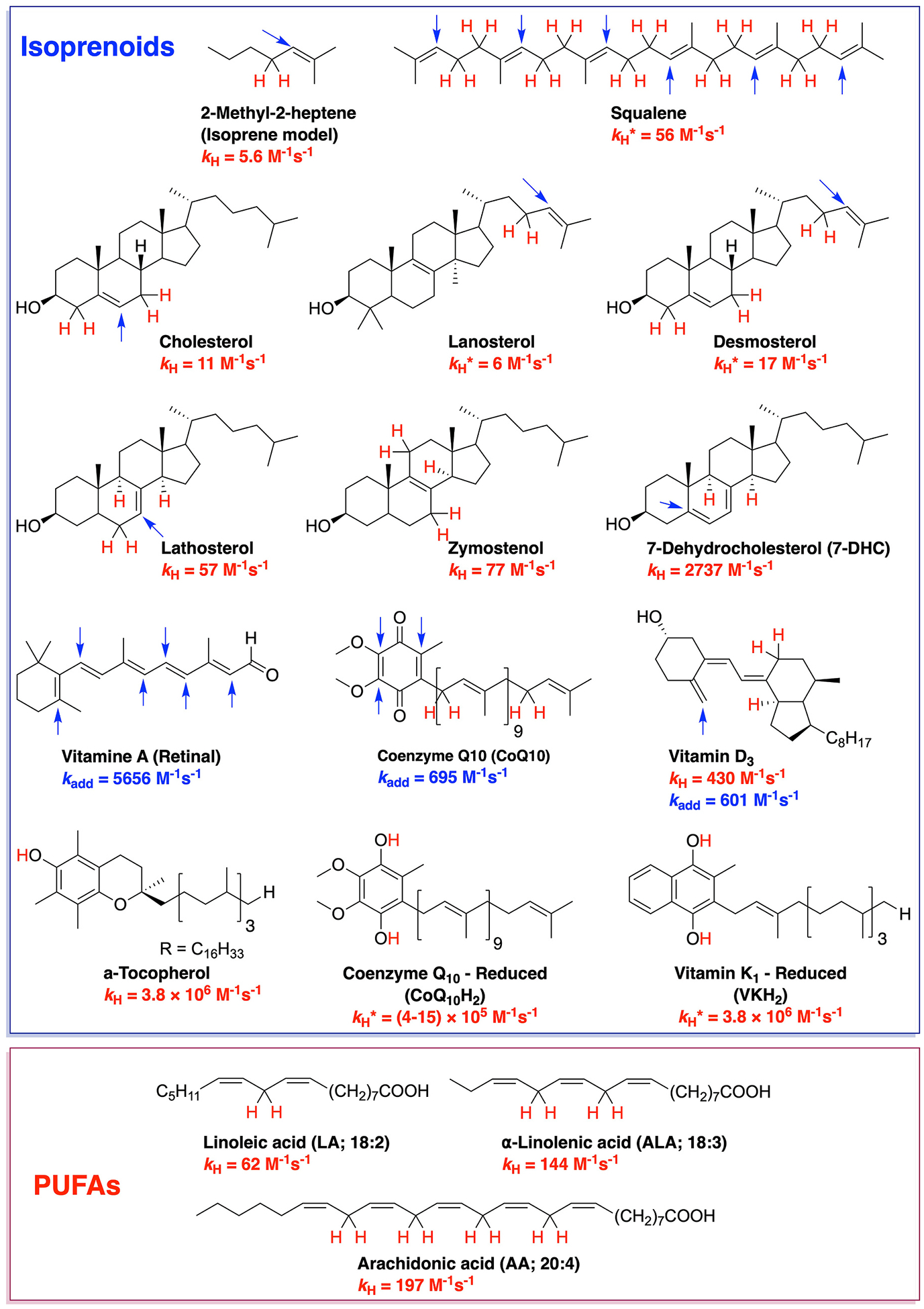
Structures of selected isoprenoid-derived lipids and polyunsaturated fatty acids (PUFAs). Reactive hydrogen atoms toward HAT are marked Red. Potential sites of PRA are marked with Blue Arrow. Available rate constants are shown under each lipid (see Table 1 for references). *, estimated rate constants based on references shown in Table 1.

The rate constants of various isoprenoid-derived lipids have been measured using the linoleate radical clock (Fig. 2 and Table 1). Cholesterol is not a highly oxidizable molecule with a *k* of 11 M^−1^s^−1^ [29], but cholesteryl ester has a larger rate constant at 31–36 M^−1^s^−1^ [22,30]. However, both are much less reactive than the least reactive PUFA, linoleic acid (LA 18:2), which has a *k*_p_ of 62 M^−1^s^−1^ [31]. On the other hand, the immediate precursor to cholesterol, 7-dehydrocholesterol (7-DHC), is extremely reactive toward peroxyl radicals, with a *k*_p_ of 2260 M^−1^s^−1^ and 2737 M^−1^s^−1^, measured in 2009 and 2021 using the two generations of the linoleate radical clock [22,29]. We suggest that the latter rate constant, 2737 M^−1^s^−1^, should be used for future studies because the new generation of radical clock accounts for both HAT and PRA rate constants, even though 7-DHC still undergoes lipid peroxidation primarily via HAT. Oxidation of 7-DHC proceeds predominantly via the HAT mechanism, as the hydrogen atoms on carbons 9 and 14 are well-positioned for abstraction by a peroxyl radical with an easily accessible transition state [29]. Other cholesterol precursors are mostly more reactive than cholesterol, with the exception of lanosterol and dihydrolanosterol. Specifically, lathosterol and zymostenol have a *k*_p_ of 57 and 77 M^−1^s^−1^, respectively [32]. The Bloch pathway cholesterol precursors all have an additional double bond in the isoprene unit on the side chain. Although the rate constants of the Bloch pathway precursors have not been directly measured, the rate constant for the isoprene unit was determined to be 5.6 M^−1^s^−1^ (in the form of 2-methyl-2-heptene) [32]. The single isoprene unit presumably predominantly undergoes HAT reaction at the secondary allylic methylene position due to smaller bond-dissociation energy and the more stabilized allylic radical with four substitutions, which can stabilize the radical through hyper-conjugation [26]. However, we cannot rule out that the isoprene unit can also undergo PRA reaction at the double bond, giving a relatively stable tertiary radical, but less stable than the allylic radical. Therefore, the *k*_p_ for these precursors, lanosterol, desmosterol, 24-dehydrolathosterol, zymosterol, and 7-dehydrodesmosterol, were estimated to be 6, 17, 63, 83, and 2743 M^−1^s^−1^, respectively [32]. Squalene is the immediate precursor to lanosterol, the first sterol, and is composed of six isoprene units. Thus, the *k*_p_ of squalene can be estimated to be 56 M^−1^s^−1^ because it has ten allylic methylene groups (compared to one in 2-methyl-2-heptene), which is more reactive than cholesterol and comparable to that of LA 18:2. More detailed discussion on the effect of various factors on the reactivity of cholesterol and its precursors and the mechanisms of their peroxidation can be found in a previous review [26].

Vitamin D_3_, or cholecalciferol, is also a highly oxidizable isoprenoid lipid, which is derived from 7-DHC upon UV irradiation, with a measured *k*_p_ of 1031 M^−1^s^−1^. However, unlike 7-DHC, it undergoes oxidation via both PRA and HAT due to the presence of a conjugated double bond system in its structure, with a *k*_add_ of 601 M^−1^s^−1^ add and a *k*_H_ of 430 M^−1^s^−1^. Vitamin A contains 6 conjugated double bonds, making it highly prone to oxidation. Indeed, its *k*_p_ was measured to be 5656 M^−1^s^−1^ with the oxidation proceeding entirely via the PRA mechanism [22]. This is the largest rate constant for lipid peroxidation that has been measured via the linoleate radical clock to date. The oxidized form of coenzyme Q_10_ (CoQ_10_), or ubiquinone, is an endogenous isoprenoid that was also found to be highly oxidizable, with a rate constant of 695 M^−1^s^−1^, proceeding entirely via PRA [22].

Endogenous phenolic isoprenoids are potent free radical-trapping antioxidants against lipid peroxidation. α-Tocopherol, the major form of vitamin E, has a *k*_H_ (*k*_inh_ to be more accurate) of 3.2 × 10^6^ M^−1^s^−1^ at 30 °C and an extrapolated *k*_inh_ of 3.8 × 10^6^ M^−1^s^−1^ at 37 °C based on measured rate constants at different temperatures [24,33,34]. The reduced form of CoQ_10_, ubiquinol (CoQ_10_H_2_), is also a radical-trapping antioxidant, with a *k*_inh_ of about 10–39 % that of α-tocopherol in homogenous solutions, depending on the solvent, but almost the same *k*_inh_ in liposomes [35,36]. The reduced form of vitamin K (also a quinone), VKH_2_, is another radical-trapping antioxidant with a similar *k*_inh_ to α-tocopherol in homogenous solutions, but four times larger *k*_inh_ in liposomes [37].

Although these isoprenoid lipids display a wide range of reactivity toward free radical oxidation, they all exert anti-ferroptotic properties based on the existing literature. Here we will examine the anti-ferroptotic effects of these isoprenoid lipids in more detail and discuss the likely protective mechanisms of each.

## 3. Phenolic isoprenoids: vitamin E, CoQ_10_H_2_, and VKH_2_

Vitamin E, CoQ_10_H_2_, and VKH_2_ have been found to act as potent ferroptosis inhibitors [12,13,38]. This is understandable because they terminate the free radical lipid peroxidation chain reactions. In fact, other endogenous and exogenous radical-trapping phenols or arylamines have been found to be effective ferroptosis inhibitors, such as ferrostatin-1 and liproxstatin-1 [1,9].

The compound CoQ_10_, or ubiquinone, is an electron carrier in the electron transfer chain. It is composed of a benzoquinone core linked to an isoprene chain and is synthesized in the mitochondria. CoQ_10_ is present in a wide variety of tissues and subcellular compartments, demonstrating its multifunctionality [39,40]. The isoprene chain of CoQ_10_ is synthesized via the mevalonate pathway, starting with acetyl-CoA and ending with farnesyl pyrophosphate, or FPP. The chain is made of ten isoprene units, and is responsible for the lipophilicity of CoQ_10_, which allows it to accumulate in cell membranes and other lipid-rich spaces [41]. The reduced form of ubiquinone, CoQ_10_H_2_, not only acts as a radical-trapping antioxidant, but also detoxifies peroxides to nontoxic alcohols by transferring electrons from NADPH [42]. CoQ_10_H_2_ has also been found to effectively regenerate vitamin E with a rate constant of 3.74 × 10^5^ M^−1^s^−1^ at 25 °C [43].

Through genetic screening, it was found that the enzyme responsible for the regeneration of the reduced CoQ_10_H_2_ from its oxidized form, CoQ_10_, acts as a ferroptosis suppressor [12,13]. The enzyme was originally called flavoprotein apoptosis-inducing factor mitochondria-associated 2 (AIFM2) and was previously shown to be a pro-apoptotic gene [44]. However, later studies showed that the enzyme does not localize to the mitochondria, nor does it promote apoptosis [12]. Therefore, it was renamed to ferroptosis suppressor protein 1, or FSP1, to reflect its newfound role in preventing lipid peroxidation. It was shown by Doll et al. that cells overexpressing FSP1 showed marked resistance to ferroptosis-inducing compounds, such as RSL3, and continued to proliferate in the presence of these compounds [12]. Bersuker et al. showed that FSP1 knockout cells displayed increased sensitivity to ferroptosis inducers, and that this effect could be ameliorated with the addition of iron chelators or radical-trapping antioxidants [13]. It was determined by both groups that these effects are not dependent on the presence of glutathione or the activity of GPX4, but rather due to the antioxidant activity of the electron-carrying compound CoQ_10_H_2_. Therefore, FSP1 is a key regulator of ferroptosis that depends on CoQ_10_, an antioxidant compound that is derived from the same mevalonate pathway as cholesterol biosynthesis. More recently, FSP1 was found to also reduce oxidized vitamin K, a naphthoquinone, to its reduced form (VKH_2_), which protects cells from ferroptosis [38].

The oxidized form of CoQ_10_ is also highly oxidizable and its oxidation proceeds predominantly via the PRA mechanism, with a measured k_p_ of 695 M^−1^s^−1^. It has been shown that the treatment of cells with exogenous CoQ_10_ decreases toxicity and lipid peroxidation associated with RSL3 and erastin treatment [20]. Although, we cannot rule out the possibility that CoQ_10_ might exert its activity after being reduced in the cells by FSP1. Taken together, these studies suggest that CoQ_10_H_2_ likely protects the cells from ferroptosis through its radical-trapping property but its oxidized form may also be able to protect the cells in a sacrificial manner by diverting free radicals away from PUFA-containing phospholipids.

## 4. 7-DHC, vitamin A, and vitamin D

7-DHC is the immediate precursor to cholesterol in the Kandutsch-Russel pathway of cholesterol biosynthesis. It is reduced to cholesterol via the enzyme 7-dehydrocholesterol reductase, or DHCR7. 7-DHC is an extremely oxidizable lipid with a *k*_p_ of 2737 M^−1^s^−1^ [22,29]. This is more than ten times higher than the peroxidation of arachidonic acid, which is a highly oxidizable PUFA [29]. Recently, in a genome-wide CRISPR screen, it was shown that *DHCR7* is an essential pro-ferroptotic gene [18,19]. Later studies confirmed that the knockout of *DHCR7* conferred resistance to ferroptosis in multiple cell lines. The resistance was lost, however, when SCD5, the enzyme that produces 7-DHC, was also knocked out. It was also shown that adding exogenous 7-DHC was able to protect cells from ferroptotic death, demonstrating that the resistance was due to the accumulation of 7-DHC, not due to any up or downstream effects of DCHR7 knockout.

Previous work has demonstrated that phosphatidylethanolamine, or PE, phospholipids are significantly oxidized during ferroptosis [45]. It was shown by Freitas et al. that while 7-DHC is highly oxidizable itself, its presence reduces the formation of oxidized PE phospholipids in cells. Phospholipid truncation products, which are closely associated with membrane permeabilization [46], were found to be significantly lower in *DHCR7* knockout cells as compared to wild type [18]. Conversely, it was shown that DHCEO, a biomarker of 7-DHC peroxidation in biological systems [47], was significantly higher in cells treated with RSL3 [18, 19]. This suggests that 7-DHC could be oxidized more rapidly than phospholipids, sparing the phospholipids from oxidation and thereby preventing ferroptotic death in a sacrificial manner.

Recently, it was also shown that the addition of vitamin D or vitamin A can attenuate cancer cell death induced by the ferroptosis inducers RSL3 and erastin [20]. As discussed above, these two lipids are also highly reactive toward peroxyl radicals but favor the PRA oxidation mechanism (Table 1). It is likely that the protective effect of vitamin D, vitamin A, and 7-DHC are due to their high propensity for oxidation with propagation rate constants significantly larger than those of PUFAs (Table 1 and discussion above). Since they are more prone to oxidation than even highly unsaturated phospholipids, they could act as free radical diverters, redirecting free radical chain reactions away from PUFA-containing phospholipids. In other words, they could protect against ferroptosis through a sacrificial mechanism. When these lipids are co-oxidized with the PUFA LA 18:2, this PUFA is mostly spared from being oxidized [22]. We speculate that the protective effect of these lipids against ferroptosis may be due to several reasons: 1) propagation to isoprenoids themselves is more favored than to PUFAs in the membrane because PUFAs are fixed to phospholipids and limited to lateral diffusion, while the less polar isoprenoids can diffuse in any direction inside the lipid membrane; 2) the isoprenoid-derived radicals might have larger termination rate constants than PUFA-derived radicals, which decreases the overall oxidation rates; 3) in biological systems, it is possible that the oxidation of these isoprenoid lipids might not cause membrane damage to the same extent as the oxidation of phospholipids because they are not required to maintain the structure of the lipid bilayers, thus preventing ferroptosis. In summary, they may protect cells from undergoing ferroptosis primarily by acting as sacrificial lipids due to their highly oxidizable structures and less membrane-damaging oxidation products.

## 5. Cholesterol and desmosterol

Cholesterol itself is not particularly prone to oxidation, with a peroxidation rate constant of 11 M^−1^s^−1^ in the free form and 36 M^−1^s^−1^ in the ester form and its oxidation proceeding primarily via HAT [22,29]. However, in a 2023 paper by Sun et al., it was shown that treatment of HT-1080 cells with 7-DHC, cholesterol, and desmosterol all attenuated cell death and lipid peroxidation induced by RSL3 treatment [15]. Unlike 7-DHC, cholesterol and desmosterol did not directly reduce lipid peroxidation in liposomes as measured by C11-BODIPY. Because cholesterol and CoQ_10_ are both synthesized via the mevalonate pathway (Fig. 4), which is sensitive to negative feedback from downstream products, the group reasoned that excess cholesterol could lead to increased production of CoQ_10_. Indeed, treatment with exogenous cholesterol and desmosterol was shown to increase intracellular CoQ_10_ content, providing a potential mechanism of the ferroptosis resistance offered by cholesterol and desmosterol [15]. In another recent study by Liu et al., cholesterol was found to regulate ferroptosis through the mTOR pathway [16]. They found that cholesterol, through SLC38A9, activates mTOR, which upregulates SLC7A11 (the cystine importer) and GPX4 and inhibits ferritinophagy, both conferring resistance to ferroptosis.

In addition to its regulatory roles, the amount of cholesterol in a cell membrane can have profound effects on its structure and function. It plays a regulatory role in many processes, including membrane fusion, permeation, transport, and protein-protein interactions [48]. While the effects of cholesterol on cellular membrane function are diverse, it is generally accepted that the presence of cholesterol within a membrane increases its rigidity, particularly in regions of high PUFA content [48]. While older studies postulated that cholesterol could act as a sacrificial antioxidant in a similar mechanism to the highly oxidizable lipids discussed in this review [48], the relatively low peroxidation rate constant of cholesterol does not support this hypothesis [29]. Indeed, it has been shown in more recent studies that the presence of cholesterol in membranes at a physiologically relevant concentration (30 mol %) can prevent the oxidation of polyunsaturated fatty acids by increasing membrane rigidity and slowing the rate at which peroxidation spreads through a lipid membrane [49]. This introduces an alternative non-sacrificial mechanism by which less oxidizable isoprenoid-derived lipids may protect cells from ferroptosis: through alteration of membrane physicochemical properties. Indeed, a recent study found that cholesterol decreased membrane fluidity and promoted lipid raft formation, which slowed the rate of lipid peroxidation [17].

## 6. Squalene

Another mechanism proposed by Sun. et al. to explain the anti-ferroptotic effect of cholesterol was the cholesterol-dependent inhibition of squalene epoxidase, or SQLE [15]. SQLE, which catalyzes the second rate limiting step in cholesterol biosynthesis, is negatively regulated by the presence of excess cholesterol [50,51]. It was shown that ablation of SQLE conferred resistance to cell death induced by RSL3, to a similar extent as cholesterol and desmosterol supplementation. This agrees with previous work completed by Garcia-Bermudez in 2019 [14], which showed that some cancer cells that rely on exogenous cholesterol for growth display significantly reduced expression of SQLE and increased resistance to ferroptotic death. Further study of these cells showed that the knockout of FDFT1, the upstream enzyme that produces squalene, abolished the protective effect of diminished SQLE expression. This suggests that the protective effect comes from the presence of squalene. Interestingly, exogenous squalene supplementation did not recapitulate the protection, suggesting that squalene must be present in specific subcellular compartments to be protective [14].

**Fig. 3.**
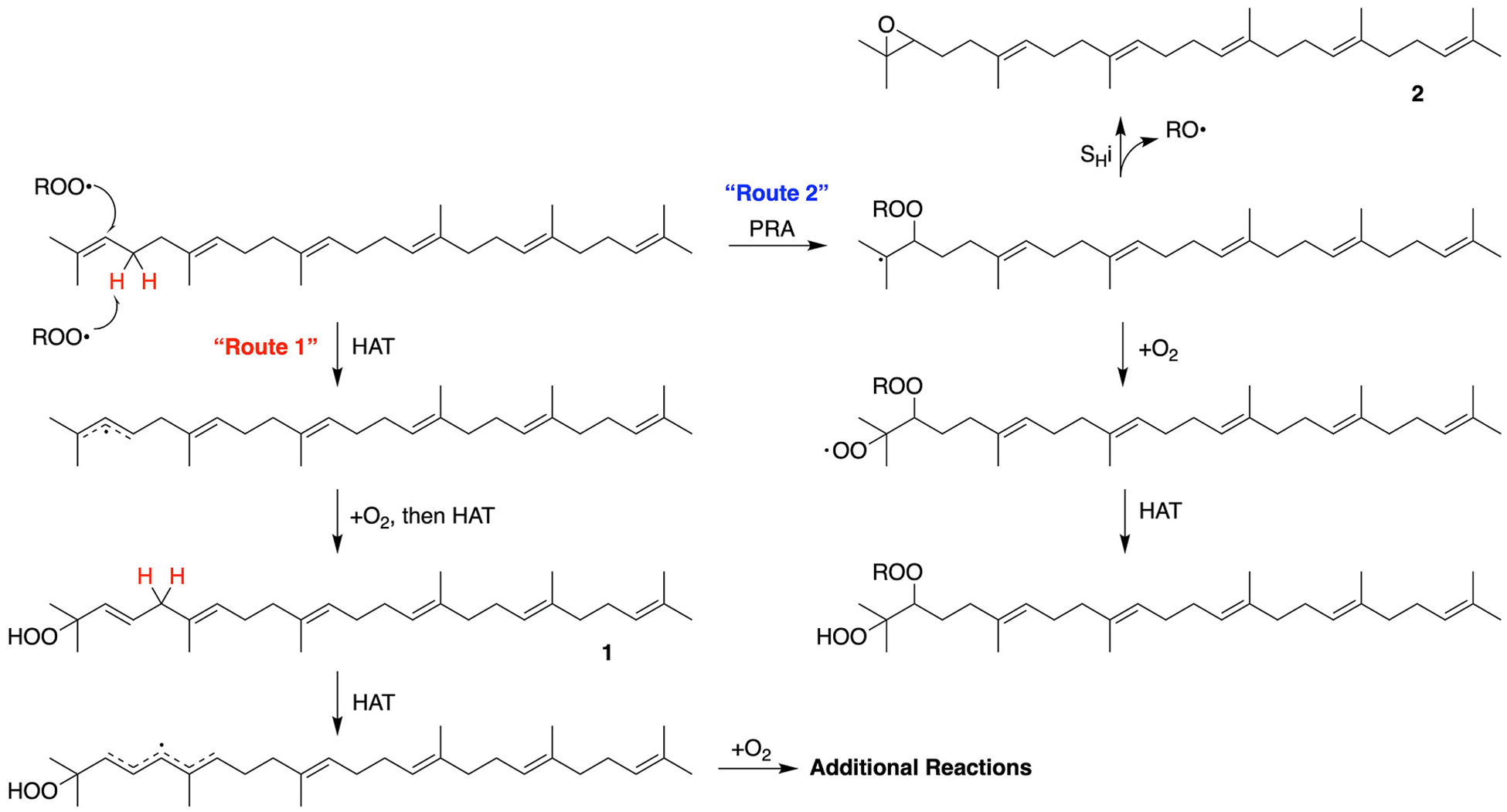
Proposed mechanisms of peroxidation of squalene via the HAT (“Route 1”) and PRA (“Route 2”) pathways using two sites as examples. Potential products from other sites of HAT and PRA (see Fig. 2) were not shown. In Route 1, initial abstraction of an allylic hydrogen atom leads to an allylic radical, which adds a molecular oxygen and then abstracts another hydrogen atom, resulting in hydroperoxide **1**. Compound **1** can undergo additional HAT reaction via the loss of the bisallylic hydrogen atoms. In Route 2, the addition of a peroxyl radical to the double bond gives a tertiary carbon radical, which can undergo S_Hi_ to give an epoxide or add another molecular oxygen to give a new peroxyl radical.

Squalene is synthesized in the endoplasmic reticulum (ER), which has been identified as an important site of lipid peroxidation in ferroptosis [52]. Its accumulation in the ER membrane may slow the progression of lipid peroxidation by increasing membrane rigidity [53]. On the other hand, the estimated propagation rate constant of squalene, 56 M^−1^s^−1^, is comparable to that of linoleic acid (62 M^−1^s^−1^), so it is likely that they could compete with the oxidation of PUFAs to some extent. While the oxidation mechanisms of cholesterol and 7-DHC have been well established [25,26,30,54,55], the mechanism for squalene oxidation has not been examined previously. However, based on its potential reactive sites, we can propose a series of oxidation products from the HAT and PRA mechanisms as shown in Fig. 3, where we use one site of PRA reactions and one site of HAT reactions as examples. As seen in the figure, initial HAT (“Route 1”) would lead to an allylic radical, which then reacts with oxygen and abstracts another hydrogen atom to give a hydroperoxide (**1**). Compound **1** contains a bisallylic methylene group that is more prone to HAT, like in a PUFA molecule, thus may undergo additional oxidation reactions. On the other hand, PRA reaction (“Route 2”) would initially lead to a tertiary radical, which can undergo S_H_i to give an epoxide (**2**) or add another oxygen molecule, giving a diperoxide after abstracting a hydrogen atom. Similar reaction pathways can be anticipated at other sites of PRA and HAT, as shown in Fig. 2. One can speculate that, due to the non-polar nature of squalene, it is likely embedded deep in the hydrophobic region of the lipid membrane prior to oxidation. However, polar functional groups were formed after squalene undergoes peroxidation, which may propel the oxidized squalene to migrate toward the aqueous-lipid interface to stabilize the polar groups with hydrogen bonding. Future studies on the formation of these products and their impact on membrane properties, such as rigidity and integrity, would shed additional light on the mechanisms of protection by squalene against ferroptosis.

## 7. Summary

**Fig. 4.**
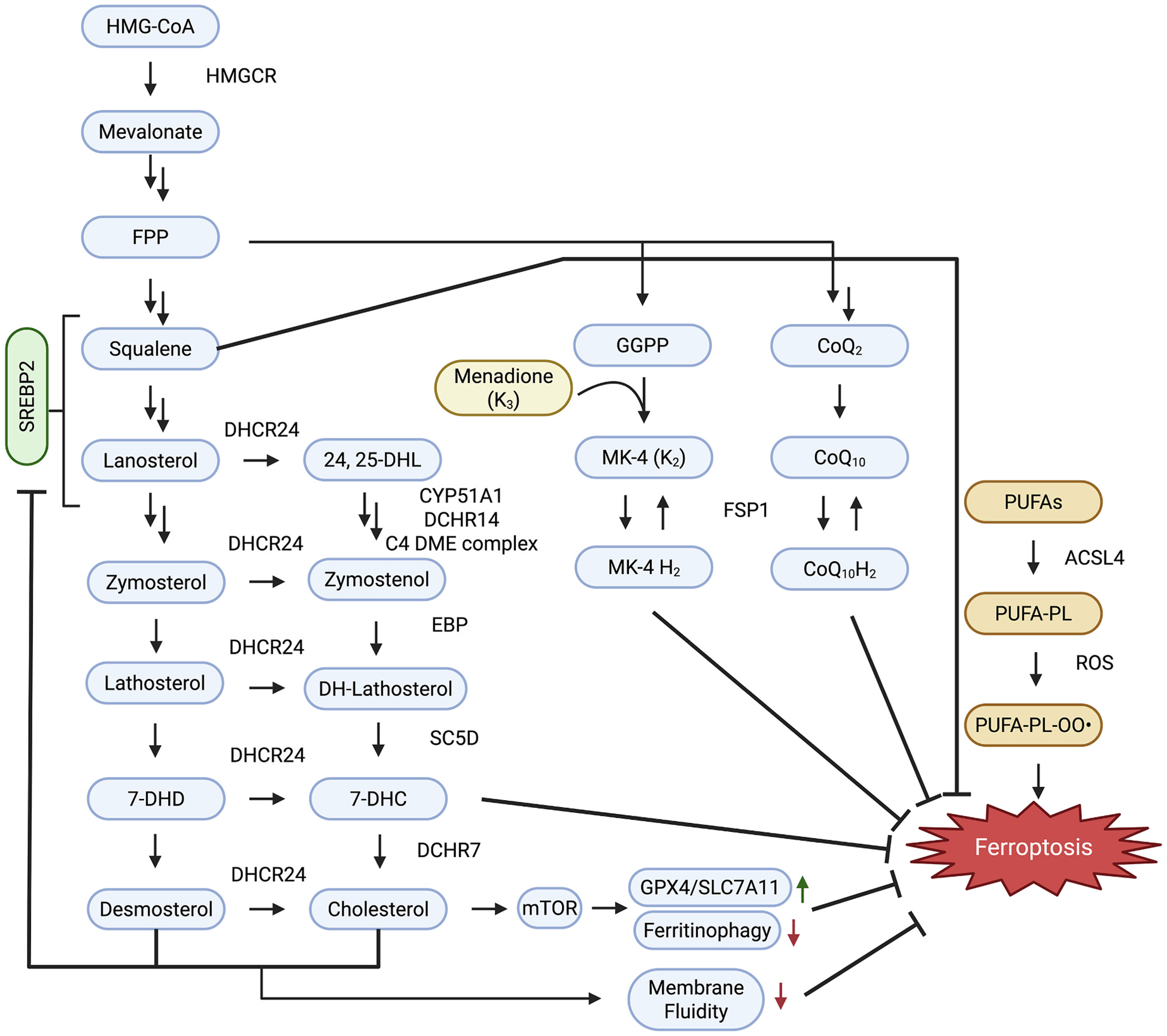
Scheme depicting the role of isoprenoid-derived lipids in regulating ferroptotic death. The left portion shows the cholesterol biosynthetic pathway starting with 3-hydroxy-3-methylglutaryl-CoA (HMG-CoA). The right portion shows the synthesis of other isoprenoids from the mevalonate pathways. HMGCR, HMG-CoA reductase; CYP51A1, cytochrome P450 51A1; DHCR14, 14-dehydrocholesterol reductase; C4 DME, C4-demethylation; EBP, emopamil binding protein or 3β-hydroxysteroid-Δ [8],Δ [7]-isomerase; SC5D, sterol-C5-desaturase; DHCR7, 7-dehydrocholesterol reductase; FPP, farnesyl pyrophosphate; DHCR24, 24-dehydrocholesterol reductase; DHL, dihydrolanosterol; DH-Lathosterol, 24-dehydrolathosterol; GGPP, Geranylgeranyl Pyrophosphate; CoQ, coenzyme Q; MK-4, menaquinone-4. This figure was created in BioRender.

Recent studies have highlighted the role of isoprenoid-derived lipids in preventing ferroptosis (Fig. 4). We suggest that the mechanism by which these lipids exert their protective effects against ferroptosis is dependent on their chemical reactivity, which can be quantified by their peroxidation propagation rate constants. Phenolic isoprenoids, such as vitamin E, VKH_2_, and CoQ_10_H_2_, exert their effect by serving as a radical-trapping antioxidants. Highly oxidizable lipids with a large propagation rate constant, including 7-DHC, vitamin D, vitamin A, and CoQ_10_, are likely to protect against ferroptosis in a sacrificial manner. Because they are more prone to oxidation than even highly unsaturated phospholipids, they are preferentially oxidized in the presence of PUFAs, thus sparing PUFA-containing phospholipids. On the other hand, lipids that are less oxidizable, including cholesterol and desmosterol, likely protect against ferroptosis in a non-sacrificial manner. Mechanisms for this type of protection are more varied, but evidence suggests that transcriptional regulation and alterations of membrane properties are two potential mechanisms of protection for cholesterol. We suggest that other cholesterol precursors, including squalene, might also protect the cells from ferroptosis using either or both mechanisms, sacrificial (for those similar to or more reactive than PUFAs) or non-sacrificial (for those less reactive than PUFAs). Understanding the ways in which cells can evade ferroptosis by modulating their lipid metabolism and the mechanism of protection or potentiation of different lipids is critical to harnessing the therapeutic potential of ferroptosis.

## Figures and Tables

**Table 1:** Propagation rate constants of selected isoprenoid-derived lipids relative to representative PUFAs in solutions at 37 °C.

Lipid	*k*_p_ (M^−1^ s^−1^)	*k*_H_ (M^−1^ s^−1^)	*k*_add_ (M^−1^ s^−1^)	Ref.
Single isoprene	5.6 ± 0.2	5.6 ± 0.2	NA^a^	[32]
Squalene	56	56	NA^a^	b
Lanosterol	6	6	NA^a^	b
Cholesterol	11 ± 2	11 ± 2	NA^a^	[29]
Cholesteryl acetate	36 ± 2	36 ± 2	NA^a^	[22]
Desmosterol	17	17	NA^a^	[32]
Lathosterol	57 ± 3	57 ± 3	NA^a^	[32]
24-dehydrolathosterol	63	63	NA^a^	[32]
Zymostenol	77 ± 5	57 ± 3	NA^a^	[32]
Zymosterol	83	83	NA^a^	[32]
Vitamin D_3_	1031 ± 63	430 ± 63	601 ± 63	[22]
7-DHC	2737 ± 83	2737 ± 83	NA^a^	[22]
Retinal (vitamin A)	5656 ± 143	NA^a^	5656 ± 143	[22]
CoQ_10_	695 ± 23	NA^a^	695 ± 23	[22]
CoQ_10_H_2_	(3.8–15.2) × 10^5^	(3.8–15.2) × 10^5^	NA^a^	c
α-Tocopherol (vitamin E)	3.8 × 10^6^	3.8 × 10^6^	NA^a^	[24, 33,34]
VKH_2_	3.8 × 10^6^	3.8 × 10^6^	NA^a^	d
Linoleic acid (LA 18:2)	62	62	NA^a^	[29]
α-Linolenic acid (ALA 18:3)	144 ± 3	144 ± 3	NA^a^	[22]
Arachidonic acid (AA 20:4)	197 ± 13	197 ± 13	NA^a^	[29]
Eicosapentaenoic acid (EPA 20:5)	249 ± 16	249 ± 16	NA^a^	[29]
Docosahexaenoic acid (DHA 20:6)	334 ± 37	334 ± 37	NA^a^	[29]

a NA: not available.

b estimated based on the rate constant of 2-methyl-2-heptene.

c estimated based on the k_H_ of α-tocopherol and references [35,36].

d estimated based on the *k*_H_ of α-tocopherol and reference [37]. Green shade: isoprenoids; Blue shade: PUFAs. *k*_p_: overall propagation rate constant; *k*_H_: HAT rate constant; *k*_add_: PRA rate constant.
